# Multivariate Optimization in the Biosynthesis of a Triethanolamine (TEA)-Based Esterquat Cationic Surfactant Using an Artificial Neural Network

**DOI:** 10.3390/molecules16075538

**Published:** 2011-06-29

**Authors:** Hamid Reza Fard Masoumi, Anuar Kassim, Mahiran Basri, Dzulkifly Kuang Abdullah, Mohd Jelas Haron

**Affiliations:** Department of Chemistry, Faculty of Science, University Putra Malaysia, 43400, Selangor, Malaysia

**Keywords:** neural network, optimization, esterquat, enzyme, synthesis

## Abstract

An Artificial Neural Network (ANN) based on the Quick Propagation (QP) algorithm was used in conjunction with an experimental design to optimize the lipase-catalyzed reaction conditions for the preparation of a triethanolamine (TEA)-based esterquat cationic surfactant. Using the best performing ANN, the optimum conditions predicted were an enzyme amount of 4.77 w/w%, reaction time of 24 h, reaction temperature of 61.9 °C, substrate (oleic acid: triethanolamine) molar ratio of 1:1 mole and agitation speed of 480 r.p.m. The relative deviation percentage under these conditions was less than 4%. The optimized method was successfully applied to the synthesis of the TEA-based esterquat cationic surfactant at a 2,000 mL scale. This method represents a more flexible and convenient means for optimizing enzymatic reaction using ANN than has been previously reported by conventional methods.

## 1. Introduction

In the past two decades, triethanolamine (TEA)-based esterquat has been the primary ingredient in European fabric softeners and is becoming the global molecule of choice for various industries [1]. To date, esterquat cationic surfactants were mainly used as textile softening agents. In particular, several investigations have been carried out during recent years that demonstrate esterquat cationic surfactants are promising ingredients in softener products, hair and cosmetic conditioner formulations [2]. In addition, they are highly biodegradable and biocompatible because their ester bonds are easily hydrolyzed [3,4,5]. Besides biodegradability additional advantages such as excellent softening properties, suitability for various fabrics and simple preparation procedures, have been discovered with the use of esterquat cationic surfactants as textile softening agents.

Chemical synthesis is the typical means to esterify triethanolamine and fatty acids. This reaction occurs in the presence of a phosphinic acid catalyst, producing various combinations of mono-, di- and tri-esteramines [6]. Esteramines are finally reacted with a quaternizing agent, such as dimethyl sulphate [(CH_3_)_2_SO_4_], to introduce the positive charge onto the molecule and thus from the esterquats. However, such a synthesis normally requires high temperatures and pressures as well as corrosion-resistant equipment, which often result in undesired by-products. The preparation of the fatty acid substrates, for example, produces many impurities giving a dark-coloured product. Subsequent downstream processes such as bleaching and distillation are needed to obtain purer and lighter-coloured products [7]. In addition, most of chemical syntheses employ toxic organic solvents as the reaction media due to their high capability of solvating the raw materials. Those toxic solvents are potentially harmful to the workers in chemical plants. The product esters containing toxic solvent residue are not permitted in the food and pharmaceutical industries. A complete removal of the solvents from products is very laborious, and is not economically feasible for industry [8]. Extra costs are incurred for the high consumption of energy, special vessels suitable for high temperatures and pressures, plant infrastructure (e.g., establishment of a power-supply plant), and facility maintenance.

Since conventional chemical synthesis has the shortcomings discussed above, researchers have been exploring alternative methods. Enzymatic preparation of fatty esters has drawn great interest for the past twenty years. Because of the bio-catalytic selectivity of enzymes, the final products contain fewer isomers and side-products [8]. Moreover, the reaction conditions of an enzymatic synthesis are considerably mild by comparison with the conventional means. Thus, reactor design is relatively simple. Also industries desire to reduce the operation and equipment expenses and to improve safety in the workplace.

In the production of TEA-based esterquat cationic surfactant by the lipase-catalyzed synthesis method there are several factors that are important to obtain high conversion yields, as the percentage of reaction conversion obtained by lipase-catalyze synthesis methods are affected by various parameters such as the amount of enzyme in the reaction, reaction time, reaction temperature, molar ratio of substrate (OA:TEA) and agitation speed. The interrelationships between the above parameters are complex, and the analysis of this chemical reduction system to optimize the factors is a time and labor-consuming work. Hence, the analyses using conventional experimental methods are inefficient.

The past decade has seen a host of data analysis tools based on biological phenomena develop into well-established modeling techniques, such as artificial intelligence and evolutionary computing. Artificial neural networks (ANNs) are now the most popular artificial learning tool in biotechnology [9]. Thus, ANNs have been shown to be a powerful tool for the optimization of multivariate parameters in a great variety of areas of interest, such as in lipase-catalyzed synthesis [9,10,11,12], fermentation media optimization [13,14,15] and in the pharmaceutical developments [16,17,18]. Artificial neural network modeling based on the multilayer perceptron (MLP) has successfully been used for multivariate analysis. A recent trend in this and other fields of research is the development of ANN models with as limited number of weights as possible, which constitutes an additional significant objective to that of minimizing the errors in the generalization set. In fact, this kind of network model will be more appropriate to avoid over training, which increases its generalization ability over a new set of data [19].

## 2. Results and Discussion

ANNs are computer programs designed to model the relationships between independent and dependent variables and are capable of modeling complex, non-linear relationships directly from the raw data. Unlike classical statistical techniques, such as response surface methodology, ANNs do not require the prior assumption of the nature of the relationships between input and output parameters, nor do they require the raw data to be transformed prior to model generation. Several neural network structures have been described [20,21]. The most common architecture is the multilayer perceptron (MLP). This network comprises an input and output layer of processing units termed nodes interconnected via one or more “hidden” layers. The number of nodes in the input and output layers is determined by the number of independent and dependent variables, respectively.

The number of hidden layer nodes is defined by the user. Model development is achieved by a process of training in which the input parameters of a set of experimental records are presented to the input layer of the network. These data are then multiplied by a weighting factor and fed forward to the nodes of the first hidden layer. The weighted outputs from the input layer are summed and transformed by a transfer function. The resultant output is weighted and fed to the subsequent layer. The output from the output layer comprises a prediction of the dependent variables of the model. Comparison of the predicted properties with the observed properties of the formulation process yields the prediction error from which a performance function (often the mean squared error) is derived. The performance function is used by a backpropagation training algorithm to adjust the weights applied at the connections between nodes in each layer. By iteratively presenting the training set to the network and adjusting the weight matrix, the performance function can be minimized. In this way, the network learns the relationships between ingredients and properties and develops a model capable of predicting the properties of any formulation/process problem lying within the model space.

There are many types of learning algorithms in the literature which can be used for training of the network [22]. However, it is so difficult to know *a priori* which learning algorithm will be more efficient for a given problem [23]. The algorithm used to train ANN in this study was Quick Propagation. This algorithm belongs to the gradient descent backpropagation algorithm class [16]. The gradient descent backpropagation algorithm is one of the most popular learning algorithms. It works by determining the output error, calculating the gradient of this error, and adjusting the ANN weights (and biases) in the descending gradient direction [24]. This algorithm includes different versions such as: Standard or Incremental backpropagation (IBP): the network weights are updated after presenting each pattern from the training data set, rather than once per iteration; Batch backpropagation (BBP): the network weights update takes place once per iteration, while all training patterns are processed through the network [25]; Quick propagation (QP) is a heuristic modification of the standard backpropagation algorithm and is very fast [26]. QP is also defined as: mixed learning heuristics without momentum, learning rate optimized during training [16].

**Table 1:** Actual and predicted values of the ANN model of TEA-based esterquat cationic surfactant synthesis.

Run No.	Enzyme Amount(w/w%)	Reaction Time (hour)	Reaction Temperature (°C)	Molar Ratio of Substrates (mole)	Agitation Speed (r.p.m.)	Conversion%	
						Actual	Predicted
Training Set							
1	5	16	60	2:1	400	56.44	51.36
2	5	16	60	2:1	137.5	47.78	44.99
3	3	24	55	1:1	550	48.22	54.60
6	3	24	65	1:1	550	63.56	63.11
11	5	16	51.25	2:1	400	39.56	37.92
13	5	16	60	2:2	400	51.11	51.36
14	3	8	55	1:1	250	44.44	43.22
17	5	30	60	2:1	400	60.00	60.26
18	7	8	55	3:1	250	25.00	27.67
20	3	8	55	3:1	250	25.56	26.36
21	5	16	60	2:1	662.5	46.22	55.09
22	7	24	65	1:1	250	63.33	63.33
24	7	8	55	3:1	550	33.78	33.41
25	8.5	16	60	2:1	400	48.44	53.85
26	5	16	60	3.75:1	400	38.00	35.86
27	7	24	55	3:1	250	35.33	38.86
30	5	16	60	2:1	400	62.44	51.36
32	5	16	60	2:1	400	58.00	51.36
33	5	16	60	2:1	400	55.33	51.36
35	3	8	65	3:1	550	44.89	47.34
36	3	24	55	3:1	250	34.89	35.22
37	7	24	65	3:1	550	67.11	62.35
39	7	24	65	1:1	550	71.33	64.32
40	1.5	16	60	2:1	400	44.22	48.65
41	7	8	65	1:1	250	48.89	55.78
43	5	16	60	2:1	400	46.89	51.36
44	5	16	60	2:1	400	52.00	51.36
47	3	8	65	3:1	250	35.78	37.32
48	5	16	60	2:1	400	53.11	51.36
49	5	2	60	2:1	400	38.00	38.91
Test Set							
4	3	8	55	1:1	550	46.44	45.55
5	7	8	65	1:1	550	57.56	58.28
7	7	8	55	1:1	250	48.89	44.31
8	3	24	65	1:1	250	62.89	62.15
9	7	8	65	3:1	550	50.67	51.19
10	3	24	55	3:1	550	44.00	44.59
12	3	8	55	3:1	550	32.00	30.58
15	3	8	65	1:1	550	57.78	56.47
19	7	8	55	1:1	550	52.67	47.29
23	3	24	55	1:1	250	52.22	53.69
28	7	24	55	1:1	550	59.11	56.27
29	3	8	65	1:1	250	54.44	54.03
31	7	24	65	3:1	250	58.00	57.47
34	3	24	65	3:1	550	64.89	60.32
38	7	24	55	1:1	250	52.67	54.77
45	3	24	65	3:1	250	54.22	54.04
46	7	8	65	3:1	250	41.33	41.31
50	7	24	55	3:1	550	49.33	48.53

In the multivariate optimization of reaction, gradient descent backpropagation algorithm in QP version was used to train neural networks. The experimental data of central composite design were divided into two sets: 30 of the data sets were used as the training set and the remaining 18 data sets were used as the test set (Table 1). The training data was used to compute the network parameters. The testing data was used to ensure robustness of the network parameters. If a network “learns too well” from the training data, the rules might not fit as well for the test of the cases in the data. To avoid this “overfitting” phenomenon, the testing stage was used to control error; when it increased, the training was stopped [27].

In order to determine the optimum number of neurons in hidden layer, a series of topologies was examined, in which the number of neurons was varied from 1 to 15. The root mean square error (RMSE) was used as the error function. Also, the coefficient of determination (R^2^) and the absolute average deviation (AAD) were used as a measure of the predictive ability of the network. Decision on the optimum topology was based on minimum error of testing. Each topology was repeated ten times to avoid any random correlation due to the random initialization of the weights [28]. 

**Figure 1:**
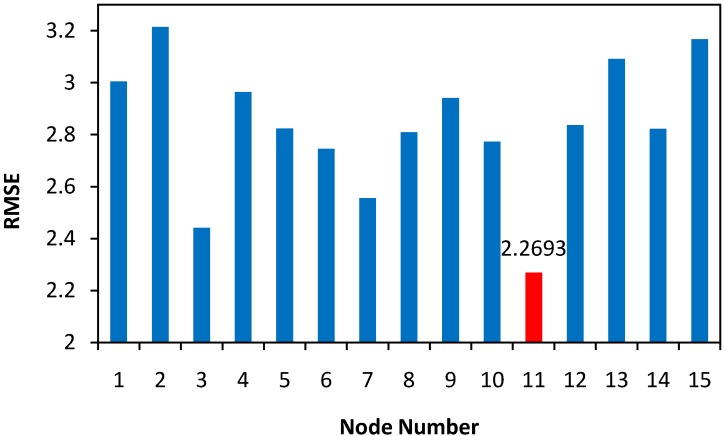
Performance of the network at different hidden nodes using QP algorithm.

**Figure 2:**
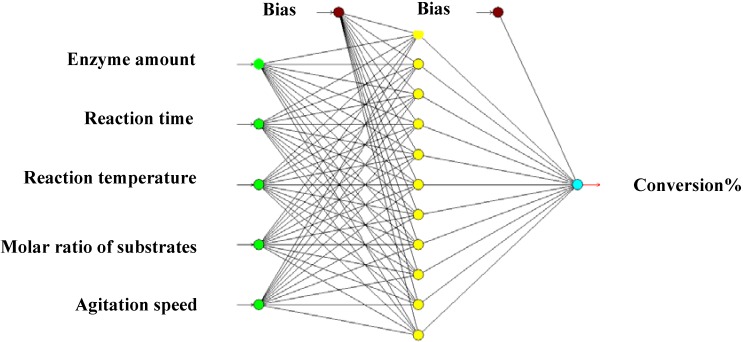
Schematic representation of a multilayer perceptron feedforward network of ANN consisting of five inputs, one hidden layer with 11 nodes and one output.

**Figure 3:**
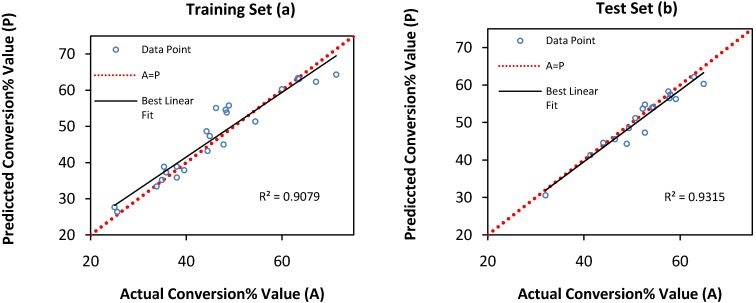
Scatter plots of predicted conversion% value *versus* actual conversion% value, (**a**) training set (**b**) test set by QP algorithm.

It was reported in literature that the quick propagation learning algorithm can be adopted for the training of all the ANN models [26]. As shown in Figure 3, the predicted model using quick propagation algorithm was fitted well to the actual values for both training and test set. However, the best results obtained with 11 hidden nodes using QP algorithm (RMSE _training set_ = 3.3834, R^2^_training set_ = 0.9079, AAD _training set_ = 6.4240, RMSE _test set_ = 2.2693, R^2^_test set_ = 0.9315, AAD _test set_ = 3.0717).

The optimal conditions for the lipase-catalyzed synthesis of TEA-based esterquat cationic surfactant were predicted as presented in Table 2 along with predicted and actual conversion% values. For this purpose, the artificial neural network based on quick propagation was adopted for predicting conversion reaction in optimal conditions using a central composite design. Experiment was then carried out under the recommended conditions and resulting response was compared to the predicted value. The optimum reaction parameters were: enzyme amount of 4.77 wt%, reaction time of 24 hours, reaction temperature of 61.9 °C, substrates molar ratio (OA:TEA) of 1:1 mol (708 mmol of OA and TEA) and agitation speed of 480 r.p.m.

**Table 2:** Optimum conditions derived by central composite design and ANN for TEA-based esterquat cationic surfactant synthesis.

Optimal Conditions					Conversion %		
Enzyme Amount (w/w%)	Reaction Time (hour)	Reaction Temperature (°C)	Molar Ratio of Substrates (mole)	Agitation Speed (r.p.m.)	Actual	Predicted	Relative Deviation%
4.77	24	61.9	1:1	480	63.57	61.14	3.98

The experimental reaction gave a reasonable percentage conversion of 63.57%. This result confirmed the validity of the model, and the experimental value was determined to be quite close to the ANN predicted value (61.14%; less of 4% in relative deviation), implying that the empirical model derived from the central composite design can be used to adequately describe the relationship between the independent variables and response. A lower amount of Novozym 435 is required to produce the respective value of product in the aforementioned experiment. In the experimental run numbers 37 and 39 higher yields were observed than under the optimum conditions even though the authors chose the optimum conditions because from the process point of view, it would be desirable to use the lowest amount of enzyme possible to achieve maximum conversion of substrate [29]. This is because Novozym 435 is more expensive that the other substrates, thus high reaction conversion with a low amount of enzyme. Moreover, shorter reaction time, lower reaction temperature and suitable range of agitation speed were considered in optimization process because longer reaction times and higher reaction temperatures lead to enzyme denaturation. Slow liquid movement would prevent the collision between enzyme and substrate, whilst an excessive agitator speed would cause shear effects on the immobilized enzyme, both of which could result in lower reaction conversion, especially with longer reaction times.

## 3. Experimental

### 3.1. Materials

Novozym 435, *Candida antarctica* lipase B immobilized on a macroporous acrylic resin (10,000 propyl laurate units per gram), was purchased from Novo Nordisk A/S (Bagsværd, Denmark). The enzyme is a granular product with a particle size of 0.2-0.6 mm. The bulk density of Novozym 435 is 350-450 kg/m^3^. *n*-Hexane obtained from J.T. Baker (USA) was used as the organic solvent. Oleic acid and triethanolamine were purchased from Merck (Germany). All other chemicals used in this study were of analytical reagent grade.

### 3.2. Methods

#### 3.2.1. Experimental Design

A 5-level-5-factor central composite design (CCD) was employed. The fractional factorial designs consisted of 32 factorial points, 10 axial points (two axial points on the axis of each design variable at a distance of 1.75 from the design center), and eight center points. The variables and their levels selected for the study were represented in Table 3.

#### 3.2.2. Enzymatic Esterification and Analysis of Samples

The reactions were performed in a 2,000 mL reactor and specified volumes of hexane were added as solvent. The reactor consisted of a screw cap and a glass flask with a capacity of 2-liter and an inner diameter of 10 cm. A four bladed impeller (4.5 cm in diameter) was immersed in the reaction mixture a 2 cm-height from the bottom of the flask to provide agitation effect. The impeller was connected by a shaft to motor for speed controlling purpose. A baffle was connected to the cap and immersed to the reaction mixture in the reactor. The reaction temperature was controlled by immersing reactor in a temperature-controlled water bath. The reactions were catalyzed by various amount of Novozym 435 from 1.5 to 8.5 wt% of oleic acid for experimental design, respectively, at different temperature (51.25-68.75 °C) and agitation speed (137.5-662.5 r.p.m.) values. The studied ranges of the substrates were 708 mmol for OA as a constant amount while concentrations of TEA were varied according Table 1 for the experimental design. All experiments were carried out in the range of 2-30 h, as showed in Table 1.

**Table 3:** Variables and their levels employed in the central composite design.

Variables		Units	Coded Level of Variables				
			−1.75	−1	0	1	1.75
X_1_	Enzyme amount	% w/w	1.5	3	5	7	8.5
X_2_	Time	Hour	2	8	16	24	30
X_3_	Temperature	°C	51.25	55	60	65	68.75
X_4_	Molar ratio of substrates	OA:TEA (mole:mole)	0.25:1	1:1	2:1	3:1	3.75:1
X_5_	Agitation speed	r.p.m.	137.5	250	400	550	662.5

At the end of the reaction periods, a 30 mL aliquot was withdrawn from the system using a syringe. The reaction sample was terminated by dilution with 10 mL of ethanol-acetone (50:50, v/v). The enzyme particles were then separated by filtration and the remaining free acid in the reaction mixture was determined by titration of the aliquots of reaction mixture against standard NaOH. The moles of acid reacted were calculated from the values obtained for the blank (without enzyme) and test samples. The ester formed was expressed as equivalent to conversion of the acid. The accuracy of the method to follow ester formation was confirmed by thin-layer chromatography (TLC) using chloroform-methanol (95:5) solvent system. 

#### 3.2.3. Artificial Neural Network

The commercial ANN software NeuralPower version 2.5 (CPC-X Software) was used throughout the study. This software allows the user to select the network type, the number of hidden layers and hidden layer neurons, the iterations used during the model training and the transfer functions. A self-organizing feature map network was used to predict the enzymatic reaction rates. The network architecture consisted of an input layer with five neurons (five variables), an output layer with one neuron (one response), and a hidden layer. To determine the optimal network topology, the number of neurons in the hidden layer was iteratively determined by developing several networks that vary only with the size of hidden layer and simultaneously observing the change in the root mean squared errors (RMSE). The transfer function was chosen sigmoid and other parameters for network were chosen as the default values of the used software. The experimental data of central composite design were used as the training and the test data of the artificial neural network. At the start of the training, weights were initialized with random values. The neural network was trained with the data obtained from 30 experimental points.

#### 3.2.4. Verification of Predicted Data

The estimation capability of ANN based on QP was tested. For this purpose, the technique was used to predict the responses at 18 experimental points. The predicted responses obtained from ANN were compared with the actual responses. The coefficient of determination (R^2^), root mean squares error (RMSE) and absolute average deviation (AAD) were determined. The RMSE and AAD are calculated by the following equations:

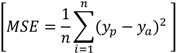
(1)

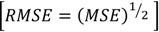
(2)

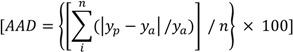
(3)
where n is the number of points, y_p_ is the predicted value obtained from the neural network model, y_a_ is the actual value.

## 4. Conclusions

A feed-forward multilayer perceptron artificial neural network based on the quick propagation model with one hidden layer and 11 neurons was successfully used to optimize the experimental conditions for the synthesis of a TEA-based esterquat cationic surfactant using the lipase-catalyzed synthesis method. Enzyme amount, reaction time, reaction temperature, molar ratio of substrates (OA:TEA) and agitation speed were chosen as main parameters. Using the best performing ANN, the optimum conditions predicted were an enzyme amount of 4.77 w/w%, reaction time of 24 h, reaction temperature of 61.9 °C, substrates molar ratio(OA:TEA) of 1:1 mole and agitation speed of 480 r.p.m. The relative deviation percentage under these conditions was less than 4%. The optimized method was successfully applied to synthesis of TEA-based esterquat cationic surfactant at a 2,000 mL scale. This paper shows that ANN model has a good potential to model non-linear and multi-variable process parameters on the reaction conversion, so that the development tasks can be performed rapidly and efficiently with an increase of productivity, consistency and quality.
